# Mental Readiness of Clients and Families Before Discharge From Drug Rehabilitation: Protocol for a Qualitative Study

**DOI:** 10.2196/89763

**Published:** 2026-08-18

**Authors:** Eny Purwandari, Aad Satria Permadi, Rini Lestari

**Affiliations:** 1 Faculty of Psychology Muhammadiyah University of Surakarta Sukoharjo, Central Java Indonesia

**Keywords:** family readiness, mental preparedness, drug rehabilitation, relapse prevention, qualitative study protocol

## Abstract

**Background:**

Relapse following drug rehabilitation remains a major mental health challenge in Indonesia. Although rehabilitation programs emphasize individual recovery, postdischarge outcomes are strongly influenced by the family as the primary microsocial environment. However, family mental readiness before discharge from rehabilitation has not been systematically explored, particularly in ways that support structured and scalable postrehabilitation care.

**Objective:**

This study aims to explore the mental readiness of clients and their families before discharge from drug rehabilitation programs and to develop a conceptual model of family readiness that can inform future scalable assessment and follow-up support strategies.

**Methods:**

This qualitative study protocol used a descriptive thematic design. Data were collected through semistructured interviews and focus group discussions with clients nearing completion of drug rehabilitation and their family members. Participants were recruited from correctional institutions and a regional mental hospital in Central Java, Indonesia. Data will be analyzed using thematic analysis following the Braun and Clarke framework. Reporting will adhere to the Consolidated Criteria for Reporting Qualitative Research (COREQ).

**Results:**

This study is funded by the Ministry of Higher Education, Science, and Technology of Indonesia under the 2025 research funding scheme. Data collection was conducted between July 2025 and December 2025, during which 28 participants were successfully recruited and interviewed. The study is structured into sequential phases, including qualitative data analysis, conceptual model development, and dissemination of findings. A manuscript reporting the initial findings was submitted to an international peer-reviewed journal in February 2026 and is currently under review. The findings are expected to generate a conceptual model of family mental readiness prior to discharge from rehabilitation, inform the development of future family-centered support strategies, and contribute to the evidence base for rehabilitation and mental health research. The final outputs of the project are expected to be completed by the end of 2026.

**Conclusions:**

By conceptualizing readiness as a relational and family-centered process, this study is expected to provide insights that can strengthen relapse prevention strategies and inform family-oriented discharge planning for drug rehabilitation systems.

**International Registered Report Identifier (IRRID):**

DERR1-10.2196/89763

## Introduction

Drug abuse remains a major public health and mental health problem in Indonesia, with relapse after rehabilitation continuing to pose a significant challenge to treatment sustainability. National reports and institutional data indicate that although structured rehabilitation programs are widely implemented, a considerable proportion of clients experience difficulties maintaining recovery after discharge [1-3]. These patterns suggest that rehabilitation outcomes are influenced not only by treatment effectiveness during institutional care but also by psychosocial conditions following discharge.

Previous studies emphasize that substance use disorders are closely associated with psychological vulnerability, emotional dysregulation, and maladaptive coping processes [4,5]. Consequently, rehabilitation programs have increasingly incorporated psychological and behavioral components alongside medical treatment [6,7]. However, most rehabilitation models remain predominantly focused on individual readiness and motivation for change, while broader ecological and relational influences receive less systematic attention [8].

Within collectivist sociocultural contexts such as Indonesia, the family constitutes the primary microsocial environment to which clients return after rehabilitation [9]. Families play a central role in providing emotional support, regulating behavior, transmitting values, and shaping daily routines during the postrehabilitation period [10]. Existing literature referenced in this study indicates that family dynamics, such as attachment quality, the level of involvement, belief systems regarding recovery, and commitment to supporting change, can function either as protective factors or as sources of stress that increase relapse risk [11,12].

Despite this evidence, family mental readiness before rehabilitation discharge has not been clearly conceptualized as a structured psychological construct. Most existing studies focus on family support during treatment or after relapse has occurred, rather than examining how families mentally prepare before clients return home [13]. This gap limits the ability of rehabilitation services to anticipate psychosocial risks and implement preventive family-centered strategies before discharge.

A qualitative exploration of mental readiness among both clients and families is therefore necessary to capture lived experiences, expectations, and relational dynamics before discharge from rehabilitation. Conceptualizing readiness as a multidimensional construct encompassing attachment, involvement, belief, and commitment provides a theoretically grounded framework for strengthening relapse prevention and informing structured assessment and follow-up planning within rehabilitation systems.

A qualitative exploration of mental readiness among both clients and families is therefore necessary to capture lived experiences, expectations, and relational dynamics before discharge from rehabilitation. Family context is widely recognized as a key determinant of psychological adjustment and behavioral outcomes, with mindful parenting, supportive family functioning, and active familial involvement contributing significantly to recovery processes and relapse prevention [10,14]. Moreover, in cases involving youth and substance-related issues, family engagement also plays an important role in ensuring protective supervision, ethical decision-making, and supportive environments that facilitate sustainable reintegration after rehabilitation [15]. Conceptualizing readiness as a multidimensional construct encompassing attachment, involvement, belief, and commitment therefore provides a theoretically grounded framework for strengthening relapse prevention and informing structured assessment and follow-up planning within rehabilitation systems [16,17].

Accordingly, this study aims to explore the mental readiness of clients and their families before completion of drug rehabilitation and to develop a contextualized conceptual model of family readiness that can support postrehabilitation care planning and future scalable interventions. Although previous studies have highlighted the importance of family involvement in substance use recovery, family readiness prior to rehabilitation discharge has not been systematically conceptualized as a psychological construct. Understanding how families mentally prepare for a client’s return home is important because expectations, emotional preparedness, and relational dynamics may significantly influence postrehabilitation adjustment and relapse risk [18].

This study therefore explores the mental readiness of both clients and their families before discharge from rehabilitation. Although previous literature suggests that relational aspects such as attachment, involvement, belief systems, and commitment may shape family responses to recovery, these elements are treated in this study as preliminary sensitizing concepts rather than predetermined categories. The final conceptual model will be derived inductively from participants’ lived experiences.

## Methods

### Study Design

This study used a descriptive qualitative design to explore the mental readiness of clients and their families prior to discharge from drug rehabilitation programs. This design was selected to capture participants’ lived experiences, perceptions, and relational processes surrounding readiness for postrehabilitation life, which are context dependent and not easily quantified [19]. A descriptive qualitative approach allows rich, straightforward accounts of experiences without imposing predetermined theoretical constructs, making it particularly suitable for protocol-driven exploratory research.

The study was designed to generate an empirically grounded understanding of mental readiness, as it is experienced and negotiated by both clients and family members. By including individual and joint perspectives, the design supports the examination of readiness as a dynamic and relational process, rather than as a static individual characteristic. This approach aligns with the study’s aim to inform the development of a conceptual framework that is relevant for practice and policy.

As a research protocol, this study emphasizes methodological transparency, reproducibility, and adherence to established qualitative research standards. Detailed reporting of the study design, participant selection, data collection, and analytic procedures is intended to facilitate replication, adaptation, and future comparative research across rehabilitation settings.

### Study Setting

The study was conducted in institutional drug rehabilitation settings in Central Java, Indonesia, including selected correctional facilities and a regional mental hospital that provided structured rehabilitation programs for individuals recovering from narcotic and psychotropic substance use. These institutions represented key service environments where clients underwent formal treatment and preparation for discharge before returning to their families and communities.

The correctional facilities included in the study offered rehabilitation programs integrated within the criminal justice system, focusing on behavioral change, psychosocial support, and reintegration planning for incarcerated individuals with substance use histories. The regional mental hospital provided clinical and psychosocial rehabilitation services for individuals with substance-related disorders referred through health or legal pathways. Together, these settings reflected diverse institutional contexts within which discharge planning and postrehabilitation transition were managed.

Conducting the study across multiple institutional settings allowed exploration of mental readiness within varying organizational structures, treatment approaches, and policy frameworks. This diversity was expected to enhance the contextual richness of the data and support the assessment of the potential transferability of findings to other rehabilitation settings with similar characteristics.

### Participants and Recruitment

Participants consisted of two groups: (1) clients undergoing drug rehabilitation who were approaching discharge and (2) family members who were expected to serve as the primary support system following discharge. A purposive sampling strategy was applied to recruit information-rich participants who were directly relevant to the study objectives [20]. Recruitment was facilitated through coordination with institutional officers.

### Eligibility Criteria

Clients were eligible to participate if they were currently enrolled in a drug rehabilitation program and were nearing completion of rehabilitation, as determined by institutional schedules and recommendations from rehabilitation staff. Eligible clients were required to be physically and psychologically stable, able to communicate their experiences clearly, and willing to participate in either individual interviews or focus group discussions. Clients also provided written informed consent prior to participation.

Clients were excluded if they had significant cognitive impairment, acute psychiatric symptoms, or other conditions that could interfere with effective communication during data collection. Clients who withdrew consent at any stage or were unable to complete the interview or discussion process were also excluded.

Family members were eligible if they were directly involved in the client’s postdischarge living arrangements, such as parents, spouses, siblings, or other close relatives who were expected to provide daily or primary support after discharge from rehabilitation. Family participants were required to be aged ≥18 years, able to communicate effectively, and willing to provide informed consent. Family members who were not involved in postdischarge care, who had limited contact with the client, or who were unable to participate in interviews or focus group discussions because of communication barriers were excluded.

### Data Collection Procedures

Data were collected using multiple qualitative techniques to enhance the depth, credibility, and triangulation of the findings. These techniques included the following: (1) focus group discussions with clients nearing discharge, (2) semistructured individual interviews with selected clients, (3) focus group discussions with family members, (4) individual interviews with selected family members, and (5) joint focus group discussions involving both clients and their family members. The combination of individual and group-based methods was intended to capture both personal experiences and shared relational dynamics relevant to mental readiness prior to discharge from rehabilitation [21].

Data collection was conducted in sequential phases. On the first day, the following activities were conducted: (1) focus group discussions were conducted with clients approaching discharge to explore shared experiences and expectations regarding postrehabilitation life, and (2) selected clients approaching discharge were invited to participate in individual semistructured interviews to explore sensitive experiences or unique perspectives in greater depth. On the second day, (1) focus group discussions were conducted with family members to explore their perceptions of readiness, anticipated responsibilities, and support strategies; and (2) purposively selected participants were invited to participate in individual semistructured interviews to explore sensitive experiences or unique perspectives in greater depth. Finally, joint discussions involving both clients and their family members were conducted to examine relational dynamics and negotiated roles following discharge from rehabilitation.

Focus group discussions were conducted to facilitate interaction among participants and to elicit collective views, shared norms, and contrasting perspectives regarding readiness and postdischarge expectations. Individual interviews were used to explore more sensitive or personal experiences that participants might have been reluctant to disclose in group settings. Joint client-family discussions focused on shared expectations, perceived challenges, and negotiated roles following discharge.

All interviews and discussions were facilitated by trained moderators and supported by observers using semistructured interview guides. This approach allowed flexibility in probing participants’ responses while maintaining consistency across data collection sessions [22]. Data collection continued until thematic saturation was reached, indicated by the absence of new themes emerging from subsequent interviews or discussions.

All sessions were conducted in private, quiet settings within the rehabilitation facilities or other agreed-upon locations to ensure participant comfort and confidentiality. With participants’ informed consent, all interviews and discussions were audio-recorded and subsequently transcribed verbatim for analysis. Field notes were taken during and after each session to capture contextual information and nonverbal cues that enriched data interpretation.

### Interview Topics

The semistructured interview guides explored mental readiness prior to discharge from drug rehabilitation across both individual and family dimensions. Topics for clients included perceived psychological preparedness, emotional regulation, confidence in maintaining recovery, and expectations regarding postdischarge roles and responsibilities. Family interviews focused on emotional attachment, communication patterns, involvement in the recovery process, beliefs about recovery and relapse, and perceived readiness to provide support.

Joint client-family discussions examined shared expectations, anticipated challenges following discharge, boundary setting, and negotiated roles within the family. These discussions aimed to identify areas of alignment and potential tension that could influence postrehabilitation adjustment and continuity of recovery.

### Data Analysis

Audio recordings will be transcribed verbatim prior to analysis. Data will be analyzed using thematic analysis following the 6-phase framework proposed by Braun and Clarke [23]: familiarization, initial coding, theme development, theme review, theme definition, and reporting. An inductive analytic approach will be applied, allowing themes to emerge from participant narratives rather than being imposed a priori. Coding and theme development will be conducted iteratively to capture patterns related to psychological readiness, family relationships, expectations, and perceived support mechanisms. Field notes recorded during interviews and discussions will be used to complement the transcripts and support contextual interpretation. Regular discussions among the research team will be conducted to review emerging themes and enhance analytic rigor.

### Trustworthiness and Rigor

To ensure rigor and trustworthiness, the study will apply established qualitative strategies, including methodological triangulation, peer debriefing, and maintenance of an audit trail [24]. Reporting of the study will adhere to the Consolidated Criteria for Reporting Qualitative Research (COREQ) to enhance methodological transparency and completeness [25].

### Ethical Considerations

This study was conducted in accordance with the Declaration of Helsinki. Ethics approval was obtained from the Health Research Ethics Committee, Faculty of Medicine, Universitas Muhammadiyah Surakarta (5811/B.2/KEPK-FKUMS/ VIII/2025). Written informed consent was obtained from all participants. Data will be anonymized and securely stored.

Because participants were recruited from correctional institutions and a mental health facility, special attention was paid to minimizing potential power imbalances. Recruitment was conducted by the research team rather than institutional authorities to ensure that participation was voluntary. Participants were informed that their decision to participate or decline would not affect their rehabilitation status, treatment access, or institutional evaluation.

All participants received detailed information about the study and provided written informed consent before participation. Interviews were conducted in private spaces to protect confidentiality. Participants were informed that they could pause or withdraw from interviews at any time without consequences. If emotional discomfort arose during discussions, participants were offered the option to stop the interview and could be referred to available counseling services within the institution if needed.

## Results

This study is funded by the Ministry of Higher Education, Science, and Technology of Indonesia under the 2025 research funding scheme. Data collection was conducted between July 2025 and December 2025, during which 28 participants were successfully recruited and interviewed. The study is structured into sequential phases, including qualitative data analysis, conceptual model development, and dissemination of findings. A manuscript reporting the initial findings was submitted to an international peer-reviewed journal in February 2026 and is currently under review. The findings are expected to generate a conceptual model of family mental readiness prior to discharge from rehabilitation, inform the development of future family-centered support strategies, and contribute to the evidence base on rehabilitation and mental health research. The final outputs of the project are expected to be completed by the end of 2026.

## Discussion

### Anticipated Findings

This qualitative study protocol addresses a critical gap in drug rehabilitation research and policy by reframing readiness for postrehabilitation life as a family-centered and relational process rather than solely as an individual attribute. Although rehabilitation systems have traditionally emphasized treatment completion and individual motivation, qualitative inquiry highlights that recovery trajectories are deeply embedded within the social and familial contexts encountered after discharge [19]. This perspective is particularly relevant for policy and service delivery in settings where relapse rates remain high despite structured institutional care.

By focusing on family mental readiness prior to discharge from rehabilitation, this study positions families as active stakeholders in relapse prevention rather than as passive support systems. The proposed domains of readiness—attachment, involvement, belief, and commitment—reflect core psychosocial capacities that shape emotional regulation, behavioral monitoring, and meaning-making during recovery [26]. Grounding these domains in participants’ lived experiences through inductive thematic analysis is expected to ensure that the resulting conceptual model will be both theoretically informed and contextually relevant [23].

From a policy standpoint, the findings of this study are expected to inform predischarge rehabilitation planning and reintegration strategies. Current policies often lack systematic mechanisms to assess family preparedness before clients return home, potentially overlooking relational stressors that undermine recovery. Integrating family readiness into discharge procedures may enable the earlier identification of psychosocial risk and guide targeted family preparation. The structured nature of the proposed readiness domains also supports translation into standard operating procedures (SOPs) and scalable assessment frameworks within rehabilitation systems.

Methodologically, the study’s design strengthens the credibility and applicability of its anticipated findings. The inclusion of multiple perspectives—clients, family members, and joint client-family discussions—allows the examination of shared expectations, negotiated roles, and relational dynamics that are often invisible in client-only assessments. The use of multiple qualitative methods, including semistructured interviews and focus group discussions, supports data triangulation and analytic rigor [24]. Thematic analysis conducted following an established framework further enhances transparency and coherence in the interpretation of the findings [23].

Although qualitative in nature, the contribution of this study is expected to extend beyond descriptive insight. Previous qualitative evidence has demonstrated that recovery outcomes are influenced not only by individual psychological characteristics, such as emotional regulation, self-awareness, and commitment to change, but also by family support, social relationships, and reintegration challenges encountered after rehabilitation. Building on these findings, the present study advances the field by examining how families prepare psychologically for the transition from institutional rehabilitation to community life [27]. Conceptualizing family readiness as a structured, multidimensional construct provides a foundation for the future development of standardized and scalable readiness assessments, including the potential integration of digitally supported follow-up and monitoring systems. Such scalability is particularly relevant for policymakers in low- and middle-income contexts, where continuity of postdischarge support is constrained by limited human resources and geographic barriers.

### Limitations

Several limitations should be considered when interpreting the anticipated findings. As a qualitative protocol, the study does not aim to establish causal relationships or evaluate intervention effectiveness. Consequently, the findings will be contextually grounded rather than statistically generalizable. However, this limitation is inherent to qualitative research and is balanced by the study’s capacity to generate conceptual clarity and depth, which are essential prerequisites for subsequent instrument development, pilot testing, and policy evaluation studies [19].

Additionally, the study is conducted within specific institutional rehabilitation settings in Central Java, Indonesia. Although this focus enhances contextual depth, variations in institutional practices and family structures may affect the transferability of the findings to other settings. To address this, the study emphasizes methodological transparency and detailed reporting in line with the COREQ, enabling readers and policymakers to assess applicability within their own contexts [25]. Variability in family roles and levels of engagement may also influence data richness; this limitation will be mitigated through purposive sampling and the inclusion of joint discussions that facilitate triangulation of perspectives.

Overall, this study protocol contributes to the advancement of evidence-informed, family-centered rehabilitation policy by providing a rigorous qualitative foundation for understanding mental readiness before discharge from rehabilitation. By linking lived experience to structured conceptualization, the study is expected to lay the groundwork for scalable assessment approaches and to strengthen the alignment among rehabilitation practice, policy planning, and long-term recovery outcomes.

### Conclusions

This study protocol outlines a qualitative approach to exploring the mental readiness of clients and their families prior to discharge from drug rehabilitation programs. By shifting the focus from individual readiness to a family-centered and relational perspective, the study addresses a critical gap in current rehabilitation practices that often overlook the psychosocial environment to which clients return after treatment.

Through an in-depth exploration of lived experiences, this study is expected to generate a contextually grounded conceptual model of family mental readiness, encompassing attachment, involvement, belief, and commitment. This model is intended to support more comprehensive relapse prevention strategies by enabling earlier identification of psychosocial risks and strengthening family preparation before discharge from rehabilitation.

Beyond its conceptual contribution, the findings of this study are anticipated to inform policy-relevant and practice-oriented applications, including the integration of family readiness assessment into predischarge rehabilitation planning. In the longer term, the proposed framework may guide the development of structured and scalable assessment tools, including digitally supported follow-up and monitoring systems, to enhance continuity of care in resource-limited settings.

By providing a rigorous qualitative foundation, this protocol contributes to evidence-informed rehabilitation policy and is expected to lay the groundwork for future instrument development, intervention studies, and policy evaluation aimed at improving sustainable recovery outcomes.

## Appendix

Multimedia Appendix 1 Peer review report from the Fundamental Research Grant Review Committee, Ministry of Higher Education, Science, and Technology of Indonesia (Kemdiktisaintek, Indonesia).

## Abbreviations

COREQ: Consolidated Criteria for Reporting Qualitative Research
SOP: standard operating procedure
