# Supplementary material for: Mental Readiness of Clients and Families Before Discharge From Drug Rehabilitation: Protocol for a Qualitative Study
Source: JMIR Res Protoc. 2026 Aug 18;15:e89763. doi: 10.2196/89763 (PMC13535308; doi:10.2196/89763)
Supplement: Supplementary file 1 [file resprot_v15i1e89763_app1.pdf]

Feedback from Reviewers

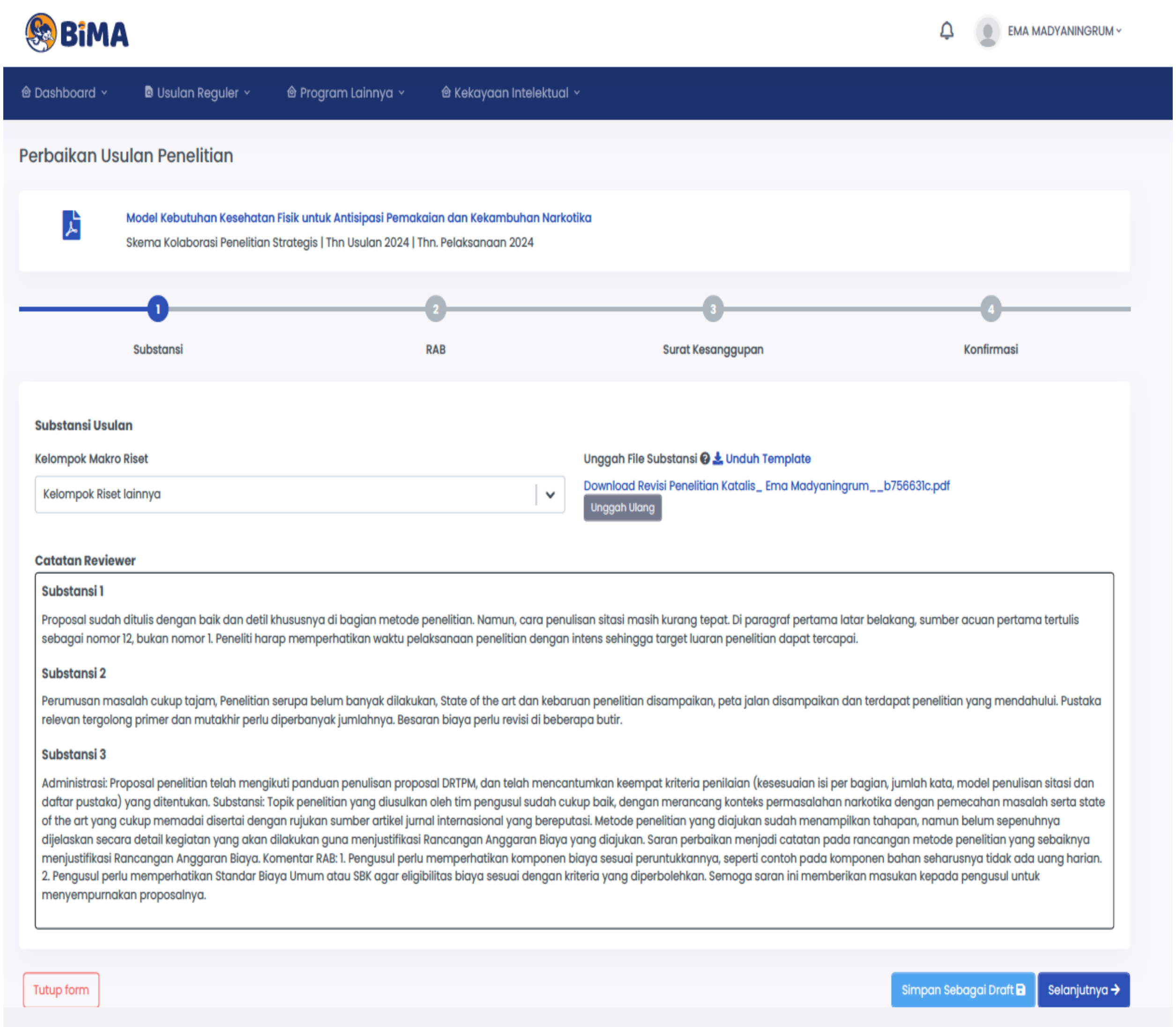

BiMA

EMA MADYANINGRUM

Dashboard | Usulan Reguler | Program Lainnya | Kekayaan Intelektual

Perbaikan Usulan Penelitian

Model Kebutuhan Kesehatan Fisik untuk Antisipasi Pemakaian dan Kekambuhan Narkotika

Skema Kolaborasi Penelitian Strategis | Thn Usulan 2024 | Thn. Pelaksanaan 2024

1 Substansi | 2 RAB | 3 Surat Kesanggupan | 4 Konfirmasi

Substansi Usulan

Kelompok Makro Riset

Kelompok Riset lainnya

Unggah File Substansi Unduh Template

Download Revisi Penelitian Katalis_ Ema Madyaningrum__b756631c.pdf

Unggah Ulang

Catatan Reviewer

Substansi 1

Proposal sudah ditulis dengan baik dan detil khususnya di bagian metode penelitian. Namun, cara penulisan sitasi masih kurang tepat. Di paragraf pertama latar belakang, sumber acuan pertama tertulis sebagai nomor 12, bukan nomor 1. Peneliti harap memperhatikan waktu pelaksanaan penelitian dengan intens sehingga target luaran penelitian dapat tercapai.

Substansi 2

Perumusan masalah cukup tajam, Penelitian serupa belum banyak dilakukan, State of the art dan kebaruan penelitian disampaikan, peta jalan disampaikan dan terdapat penelitian yang mendahului. Pustaka relevan tergolong primer dan mutakhir perlu diperbanyak jumlahnya. Besaran biaya perlu revisi di beberapa butir.

Substansi 3

Administrasi: Proposal penelitian telah mengikuti panduan penulisan proposal DRTPM, dan telah mencantumkan keempat kriteria penilaian (kesesuaian isi per bagian, jumlah kata, model penulisan sitasi dan daftar pustaka) yang ditentukan. Substansi: Topik penelitian yang diusulkan oleh tim pengusul sudah cukup baik, dengan merancang konteks permasalahan narkotika dengan pemecahan masalah serta state of the art yang cukup memadai disertai dengan rujukan sumber artikel jurnal internasional yang bereputasi. Metode penelitian yang diajukan sudah menampilkan tahapan, namun belum sepenuhnya dijelaskan secara detail kegiatan yang akan dilakukan guna menjustifikasi Rancangan Anggaran Biaya yang diajukan. Saran perbaikan menjadi catatan pada rancangan metode penelitian yang sebaiknya menjustifikasi Rancangan Anggaran Biaya. Komentar RAB: 1. Pengusul perlu memperhatikan komponen biaya sesuai peruntukkannya, seperti contoh pada komponen bahan seharusnya tidak ada uang harian. 2. Pengusul perlu memperhatikan Standar Biaya Umum atau SBK agar eligibilitas biaya sesuai dengan kriteria yang diperbolehkan. Semoga saran ini memberikan masukan kepada pengusul untuk menyempurnakan proposalnya.

Tutup form | Simpan Sebagai Draft | Selanjutnya

2025 © DRPM Diktisaintek

Translate:

Substance 1

The proposal is well written and detailed, particularly in the research methods section. However, the citation style is still inaccurate. In the first paragraph of the background section, the first referenced source is labelled as number 12 instead of number 1. The researcher is expected to pay close attention to the research timeline to ensure that the targeted outputs can be achieved.

Substance 2
The problem formulation is sufficiently precise. Similar studies have not been widely conducted. The state of the art and research novelty are clearly presented, a roadmap is provided, and prior related research is acknowledged. The number of relevant primary and up-to-date references should be increased. Several budget items require revision.

Substance 3
Administration: The research proposal adheres to the DRTPM proposal writing guidelines and includes the four required assessment criteria (section-by-section content conformity, word count, citation style, and references).
Substance: The proposed research topic is adequate, framing narcotics-related issues with appropriate problem-solving approaches and a sufficiently developed state of the art, supported by reputable international journal sources. The proposed research method outlines the stages involved, but does not fully describe the specific activities to be conducted, which are necessary to justify the submitted budget plan. Suggestions for improvement should therefore focus on refining the methodology to better justify the proposed budget.

Comments on the Budget Plan:

1. The proponents should ensure that each budget component aligns with its intended category; for example, daily allowances should not appear under materials.
2. The proponents must refer to the General Cost Standards (SBK) to ensure that all expenses comply with the eligibility criteria.

.
